# Supplementary material for: Competing instabilities reveal how to rationally design and control active crosslinked gels
Source: Nat Commun. 2022 Oct 29;13:6465. doi: 10.1038/s41467-022-34089-9 (PMC9617906; doi:10.1038/s41467-022-34089-9)
Supplement: Supplementary file 1 — Supplementary information [file 41467_2022_34089_MOESM1_ESM.pdf]

Supplementary Information

Competing instabilities reveal how to rationally design and control active crosslinked gels

Bibi Najma[1,*], Minu Varghese[1,2,*], Lev Tsidilkovski[1], Linnea Lemma[1,3,4], Aparna Baskaran[1], and Guillaume Duclos[1]

[1]Department of Physics, Brandeis University, Waltham, MA 02453
[2]Department of Physics, University of Michigan, Ann Arbor, MI 48109
[3]Department of Physics, University of California at Santa Barbara, Santa Barbara, CA 93106
[4]Current address: Department of Chemical and Biological Engineering, Princeton University, Princeton, NJ 08544
[*]These authors contributed equally

Contents

1 Hydrodynamic Theory **2**

2 Mapping between experimental and theoretical parameters **3**
2.1 Activity . . . 4
2.2 Shear and bend moduli . . . 4
2.3 Nematic elasticity . . . 5
2.4 Fitting parameters . . . 5

3 Parameter Estimation through Markov Chain Monte Carlo **5**
3.1 Fitting algorithm . . . 5
3.2 Range of fit parameters . . . 6
3.2.1 r_1 . . . 6
3.2.2 r_2 . . . 7
3.2.3 r_3 . . . 7
3.2.4 p_0 . . . 7
3.3 2D vs. 3D parameter values . . . 7
3.4 Estimation of μ_0 . . . 7
3.5 Comparison of the value of ζ with values from the literature . . . 7

4 Materials and Methods **8**
4.1 Protein purification protocols . . . 8
4.1.1 Microtubules (MTs) . . . 8
4.1.2 Crosslinkers - PRC1-NSΔC . . . 8
4.1.3 Molecular motors - K401 and K365 motors . . . 8
4.1.4 Light activable motors: K365-iLID and K365-micro . . . 8
4.2 Assembling 3D active network . . . 8
4.3 Estimation of the ratio of motor clusters and crosslinkers per microtubules . . . 9
4.4 Controlling the length distribution of microtubules via end-to-end annealing . . . 9

4.5 Flow chamber assembly . . . 9
4.6 Microscopy . . . 10
4.6.1 Widefield microscopy . . . 10
4.6.2 Confocal microscopy . . . 10
4.7 Light-activable motors: microscopy Protocol and light intensity measurement . . . 10
4.8 Image Processing . . . 10
4.8.1 Blur segmentation algorithm . . . 10
4.8.2 In- plane flow measurement . . . 11
4.8.3 Measurement of the in-plane instability wavelength . . . 11
4.8.4 Measurement of the out of plane instability wavelength . . . 11
4.8.5 Measurement of the microtubules' length distribution . . . 11

5 Supplementary Figures 12

6 Supplementary Video Captions 24

1 Hydrodynamic Theory

Consider a collection of rod-like units (microtubule filaments in the experiments) in the xy plane, all of which are initially aligned along the x-axis. Due to excluded volume interactions, there is a Frank free energy associated with distortions about this nematically ordered state, given by [1]

$$\mathscr{F}_{\text{nematic}} = \frac{1}{2}\int dx\ dy\ K\ |\vec{\nabla}\hat{n}|^2 \tag{1}$$

where K is the Frank elastic constant and $\hat{n}$ is the nematic director which represents the local orientation of the rod-like units, with $\hat{n}(t = 0) = \hat{n}_0 = \hat{x}$. Suppose that the rod-like units are cross-linked while in this configuration (by PRC1 or KSA in the experiments). The cross-linking turns the collection of rods into an elastic sheet whose undeformed state corresponds to the initial configuration of the system. Let the initial configuration of the sheet (i.e., the position of material points on the sheet) be $\vec{R}_0(x, y) = x\hat{x} + y\hat{y}$, and its current configuration be $\vec{R}(x, y) = \vec{R}_0(x, y) + \vec{u}(x)$, where $\vec{u} = u_x\hat{x} + u_y\hat{y} + h\hat{z}$ is assumed to be small. We have made the simplifying assumption of $\vec{u}$ being purely a function of x based on experimental observations. The elastic cost of the deformation,

$$\mathscr{F}_{\text{elastic}} = \frac{1}{2}\int dxdy\ \left[\nu(\partial_x u_x)^2 + \mu(\partial_x u_y)^2 + \kappa(\partial_x^2 h)^2\right] \tag{2}$$

where μ is the shear modulus, ν is a modified bulk modulus, and κ is a modified bending modulus (modified from their isotropic values due to the presence of nematic order) [1].

The nematic director in the deformed state is $n^\alpha = \frac{\partial R^\alpha}{\partial R_0^\beta} n_0^\beta$, or $\hat{n} = \hat{n}_0 + \hat{n}_0.\vec{\nabla}\vec{u} = (1 + \partial_x u_x)\ \hat{x} + \partial_x u_y\ \hat{y} + \partial_x h\ \hat{z}$ [2]. Thus, the total free energy associated with a material deformation is

$$\begin{aligned}\mathscr{F}[u_x, u_y, h] &= \mathscr{F}_{\text{nematic}} + \mathscr{F}_{\text{elastic}} &(3)\\ &= \frac{1}{2}\int dxdy\ \left[\nu(\partial_x u_x)^2 + \mu(\partial_x u_y)^2 + \kappa(\partial_x^2 h)^2 + K[(\partial_x^2 u_y)^2 + (\partial_x^2 h)^2]\right] &(4)\end{aligned}$$

[1] Starting with $\mathscr{F}_{\text{elastic}} = \frac{\tau}{2}\int dA\ C_{ijkl}u_{ij}u_{kl} + \kappa_{ijkl}H_{ij}H_{kl}$, where $u_{ij} = \frac{1}{2}[\partial_i u_j + \partial_j u_i + (\partial_i h)(\partial_j h)]$, $H_{ij} = \frac{\partial_i\partial_j h}{\sqrt{1+(\nabla h)^2}}$, and $C_{ijkl} = \chi Q_{ij}Q_{kl} + \gamma(Q_{ij}\delta_{kl} + \delta_{ij}Q_{kl}) + \lambda\delta_{ij}\delta_{kl} + \mu(\delta_{ik}\delta_{jl} + \delta_{il}\delta_{jk})$, $\kappa_{ijkl} = \alpha Q_{ij}Q_{kl} + \beta(Q_{ij}\delta_{kl} + \delta_{ij}Q_{kl}) + (\kappa' + \bar{\kappa})\delta_{ij}\delta_{kl} - \frac{1}{2}\bar{\kappa}'(\delta_{ik}\delta_{jl} + \delta_{il}\delta_{jk})$ with $\boldsymbol{Q} = S\left(\hat{x}\hat{x} - \frac{\boldsymbol{I}}{2}\right)$ the initial nematic order, $\mathscr{F}_{\text{elastic}}$ reduces to equation (2) under the assumption that $\vec{u}$ is a function of x only, where $\nu = \mu + \lambda + \gamma S + \chi S^2$, and $\kappa = \frac{1}{2}\bar{\kappa} + \kappa' + \beta S + \alpha S^2$

[2] In the neoclassical theory of liquid crystal elastomers, this relationship can be obtained under the assumption of strong crosslinking and large nematic order at the time of crosslinking [2]

Now, suppose that the material has extensile activity (resulting from ATP driven kinesin motors sliding microtubule bundles in the experiments). This results in an active force density [3],

$$\vec{f} = -\zeta \vec{\nabla} \cdot (\vec{n}\vec{n}) = -\zeta(\partial_x^2 u_y \hat{y} + \partial_x^2 h \hat{z}) \tag{5}$$

where $\zeta > 0$ is proportional to the rate at which the microtubules slide past each other. Assuming a viscous drag between the membrane and the (quasistatic) ambient medium, the rate of energy dissipation due to friction is

$$\mathscr{R} = \frac{\gamma}{2} \int dxdy\ (\partial_t \vec{u})^2 \tag{6}$$

Balancing the conservative, active, and frictional forces,

$$\frac{\delta \mathscr{F}}{\delta u_i} + \frac{\partial \mathscr{R}}{\partial \dot{u}_i} + f_i^a = 0 \tag{7}$$

i.e.,

$$\partial_t u_x = \frac{\nu}{\gamma} \partial_x^2 u_x, \quad \partial_t u_y = \frac{1}{\gamma} \left[(\mu - \zeta)\partial_x^2 - K\partial_x^4 \right] u_y, \quad \partial_t h = -\frac{1}{\gamma} \left[(K + \kappa)\partial_x^4 + \zeta \partial_x^2 \right] h \tag{8}$$

In Fourier space,

$$\partial_t \tilde{u}_x = -\frac{\nu}{\gamma} q_x^2 \tilde{u}_x, \quad \partial_t \tilde{u}_y = \frac{1}{\gamma} \left[(\zeta - \mu) q_x^2 - K q_x^4 \right] \tilde{u}_y, \quad \partial_t \tilde{h} = \frac{1}{\gamma} \left[\zeta q_x^2 - (K + \kappa) q_x^4 \right] \tilde{h} \tag{9}$$

In-plane instability The growth rate of an in-plane instability with wavenumber q_x is $\frac{1}{\gamma}\left[(\zeta - \mu)q_x^2 - Kq_x^4\right]$. Note that this expression is negative for all q_x (i.e., there is no in-plane instability) if $\zeta < \mu$, while it is positive for $q_x < \sqrt{\frac{\zeta - \mu}{K}}$ when $\zeta > \mu$. The fastest growing wave mode corresponds to $q_x = \sqrt{\frac{\zeta-\mu}{2K}}$, so that the wavelength observed in experiments should be $2\pi\sqrt{\frac{2K}{\zeta-\mu}}$. When the in plane instability exists, the growth rate of the fastest growing mode is $\frac{(\zeta-\mu)^2}{4\gamma K}$

Out-of-plane instability The growth rate of an out-of-plane instability of wavenumber q_x is $-\frac{1}{\gamma}\left[(K+\kappa)q_x^4 - \zeta q_x^2\right]$. This expression is always positive for $q_x < \sqrt{\frac{\zeta}{K+\kappa}}$. The fastest growing wavemode corresponds to $q_x = \sqrt{\frac{\zeta}{2(K+\kappa)}}$, so the experimentally observed out-of-plane wavelength should be $2\pi\sqrt{\frac{2(K+\kappa)}{\zeta}}$. The growth rate of the fastest growing mode is $\frac{\zeta^2}{4\gamma(K+\kappa)}$

Transition from out of plane to in plane instability At small activities ($\zeta < \mu$), the instability is purely out of plane. For $\zeta > \mu$, in-plane instability has a growth rate of $\frac{(\zeta-\mu)^2}{4\gamma K}$, while out of plane instability has a growth rate of $\frac{\zeta^2}{4\gamma(K+\kappa)}$. Thus, the transition from out of plane to in plane instability happens when $\left(1 - \frac{\mu}{\zeta}\right)^2 (1 + \frac{\kappa}{K}) - 1 = 0$, or $\frac{\zeta}{\mu} = \frac{1}{1 - \frac{1}{\sqrt{1+\frac{\kappa}{K}}}}$

2 Mapping between experimental and theoretical parameters

In this section, we estimate the phenomenological constants that appear in the hydrodynamic theory based on simple phenomenological models.

2.1 Activity

The chemical reaction that generates extensile activity corresponds to hydrolysis of an ATP molecule that is bound to a kinesin motor cluster (called KSA in what follows)[4]

$$\text{ATP+KSA} \underset{k_-}{\overset{k_b}{\rightleftharpoons}} \text{ATP.KSA} \xrightarrow{k_h} \text{ADP.KSA + P} \tag{10}$$

where $k_b = 2\mu M^{-1}s^{-1}$ [5, 6], $k_- = 200s^{-1}$ and $k_h = 100s^{-1}$[5, 6, 7, 8]. We have assumed there is an excess of microtubules/tubulin compared to KSA([tubulin]=13.3uM, [KSA]$\leq$ 400nM), so that almost all kinesin motors are attached to microtubules. In what follows, we will also assume that there is an excess of ATP compared to KSA (1$\leq$[ATP]$\leq$ 100μM), so that almost all the ATP in the system exist in their free state (rather than bound to KSA). Both these assumptions hold very well for the experimental system, since it is prepared with micro molar concentrations of tubulin and ATP, compared to nano molar concentration of KSA. There is an additional step in the ATP cycle, where ADP is converted to ATP by the action of an enzyme. However, this reaction is so fast that there is very little ADP in the system at any time. The rate of change in concentration of the ATP. KSA complex is given by

$$\frac{d}{dt}[\text{ATP.KSA}] = k_b[\text{ATP}][\text{KSA}]_0 - k_h[\text{ATP.KSA}] - k_-[\text{ATP.KSA}] \tag{11}$$

where $[\text{KSA}]_0$ represents the concentration of KSA that is not attached to ATP, and is given by $[\text{KSA}]_0$=[KSA]$-$[ATP.KSA] where [KSA] is the amount of KSA used to prepare the solution, and we have made the approximation $[\text{ATP}]_0 \approx$[ATP]. At steady state,

$$k_b[\text{ATP}][\text{KSA}]_0 = (k_h + k_-)[\text{ATP.KSA}] \tag{12}$$

$$\Rightarrow k_b[\text{ATP}]\Big([\text{KSA}] - [\text{ATP.KSA}]\Big) = (k_h + k_-)[\text{ATP.KSA}] \tag{13}$$

so that

$$[\text{ATP.KSA}] = \frac{[\text{ATP}][\text{KSA}]}{\frac{k_h+k_-}{k_b} + [\text{ATP}]} \tag{14}$$

Extensile activity results from the relative sliding of microtubules past one another. We therefore argue that is should depend linearly on the number of steps exerted by the ATP-bound motor clusters walking on the microtubules, hence, the concentration of ATP bound motor clusters [ATP.KSA]:

$$\zeta = \zeta_0[\text{ATP.KSA}] \tag{15}$$

where ζ_0 is a proportionality constant that depends on the efficiency with which ATP hydrolysis translates to mechanical motion.

2.2 Shear and bend moduli

There are two kinds of cross-linkers that are present in the system:

1. Non-motile kinesin motors: The concentration of these are given by $[\text{KSA}]_0$=[KSA]$-$[ATP.KSA]

2. PRC1 cross-linkers: As we did for KSA, we assume that there is an excess of tubulin compared to PRC1 so that almost all PRC1 used to prepare the solution can be assumed to be bound to microtubules.

Thus, the total concentration of cross-linkers is given by [cross-linker]=[KSA]$-$[ATP.KSA]+p0.[PRC1]. PRC1 being a sensitive protein to purify, we considered that only a fraction p0 of the proteins are

active. These cross-linkers generate elasticity in the network by constraining the motion of parts of the network relative to each other.

How the mechanical properties of purified cytoskeletal networks vary with the concentration of crosslinking proteins has been studied for a number of systems. Above a critical concentration of crosslinking proteins, the elastic plateau modulus has been found to scale with the concentration of crosslinkers, c, as $G_0 \sim c^{\gamma}$. Reported values for γ vary for different crosslinkers, and frequently fall within the range $0.4 \leq \gamma \leq 2$ [9, 10, 11, 12, 13]. While such scalings have been measured for a number of different actin crosslinking proteins, microtubule networks have been less explored. As microtubules are much more rigid than actin filaments, we here use the result from percolation theory: the elastic moduli should scale quadratically with the cross linker concentration at low cross linker concentrations[14, 9, 15, 16]. Therefore, we postulate that the shear modulus and the bend modulus should be of the form

$$\mu = \mu_0[\text{cross-linker}]^2 \tag{16}$$

$$\kappa = \kappa_0[\text{cross-linker}]^2 \tag{17}$$

We also tried to model the elastic moduli as $\sim [\text{cross-linker}]^2/(c_0^2 + [\text{cross-linker}]^2)$, where the saturating cross-linker concentration c_0 is another fitting parameter. However, we found that such a c_0 $2.2uM$, which is at least 5 times larger than our experimental concentration range, and was not a robust fitting parameter. We therefore present results for the quadratic dependence postulated above.

2.3 Nematic elasticity

Since the instability in the experiment consist of bend deformations (gradients in the deformation are along the initial direction of order, $\hat{x}$), it is appropriate to assume that K scales with the length l of microtubules as $K \sim l^3$, as is appropriate for the bend constant of liquid crystals [17].

$$K = K_0 l^3 \tag{18}$$

2.4 Fitting parameters

Using the expressions for μ, κ, K, and ζ in the equation for phase boundary,

$$\left(\frac{\zeta_0}{\mu_0}\frac{[\text{ATP.KSA}]}{[\text{cross-linker}]^2}\right)\left(1-\frac{1}{\sqrt{1+\frac{\kappa_0}{K_0}\frac{[\text{cross-linker}]^2}{l^3}}}\right)=1 \tag{19}$$

Similarly, the out of plane wavelength is given by

$$\lambda_{\text{OP}} = 2\sqrt{2}\pi\sqrt{\frac{K_0}{\zeta_0}}\sqrt{\frac{l^3}{[\text{ATP.KSA}]}\left(1+\frac{\kappa_0}{K_0}\frac{[\text{cross-linker}]^2}{l^3}\right)} \tag{20}$$

Therefore, the fitting parameters are the three ratios, $r_1 = \frac{\zeta_0}{\mu_0}$, $r_2 = \frac{K_0}{\kappa_0}$, $r_3 = \sqrt{\frac{K_0}{\zeta_0}}$, and p0, the ratio of active PRC1 proteins.

3 Parameter Estimation through Markov Chain Monte Carlo

3.1 Fitting algorithm

We assume that all data points that have a blurriness value $B < B_0$ correspond to in plane instability, and those with $B > B_0$ correspond to out of plane instability (we chose $B_0 = 0.1$ based on experimental observations). In order to fit the experimental data to the theoretical model,

we define a phase parameter $P_e = \Theta(B - B_0)$, where Θ is the Heaviside step function. P_e is unity when the instability is out of plane, and vanishes when the instability is in plane. The corresponding theoretical prediction for the phase parameter is given by $P_t = \Theta(\frac{\zeta}{\mu} - \frac{1}{1-\frac{1}{\sqrt{1+\frac{\kappa}{K}}}})$. For a given data point in the phase diagram, and a proposed set of parameters r_1, r_2, the quantity $|P_e - P_t|$ vanishes if the theoretical prediction is correct, and is equal to unity otherwise. The number of data points that the theory correctly predicts is $n = \sum_{i=1}^{N} |P_e(i) - P_t(i)|$, where N is the total number of data points from all four phase diagrams. Suppose we expect the theoretical model to predict each data point correctly with probability p (p is a measure of the uncertainty in the experimental phase boundary. We chose $p = 0.99$). The likelihood that a given set of parameters correctly predicts all four experimental phase diagrams $\sim p^n(1-p)^{N-n}$. Further, we assume that the probability that a set of parameters r_2, r_3 correctly predicts the experimental wavelengths $\sim \Pi_{j=1}^{M} e^{-\frac{(\lambda_e(j)-\lambda_t(j))^2}{(4\sigma_\lambda^2)}}$, where λ_e is the experimentally measured wavelength, λ_t is the corresponding theoretical wavelength predicted by the parameter set, σ_λ is the standard deviation of the experimental wavelength measurement, which we took to be $50\mu m$, and M is the number of data points for wavelength. Thus, the total likelihood that a set of parameters r_1, r_2, r_3 correctly predicts experimental data $\sim p^n(1-p)^{N-n}\Pi_{j=1}^{M} e^{-\frac{(\lambda_e(j)-\lambda_t(j))^2}{(4\sigma_\lambda^2)}}$.

The parameters were estimated by performing Markov chain Monte Carlo moves in the estimated parameter range. During each iteration, a new set of parameters were proposed by picking parameter values from a uniform distribution over the estimated range. If the likelihood associated with the proposed set of parameters is higher than that for the old set of parameters, the parameters were updated with the newly proposed values. If the likelihood associated with the proposed set of parameters is lower than that for the old set of parameters, a uniform random number between 0 and 1 was generated, and the new parameters were accepted only if the ratio of likelihood associated with the new parameters to that of the old parameters was larger than the random number. We started our simulations both from the upper and the lower limits of the estimated parameter range. In all cases, the Markov chain converged to the set of parameters that we report in Fig. S11. We chose the set of parameters with the maximum likelihood:

$$r1 = 4810nM$$
$$r2 = 7.02nM^2.\mu m^{-3}$$
$$r3 = 0.602\mu m^{-1/2}.nM^{1/2}$$
$$p0 = 0.592$$

We therefore estimate that only 59% of the PRC1 is actually crosslinking the microtubules.

3.2 Range of fit parameters

In order to specify a range from which parameters are picked in the Markov Chain Monte Carlo method, we need to estimate the plausible range of parameter values, given the theoretical phase boundary and the range of experimental concentrations.

3.2.1 r_1

The in-plane instability exists only for $\zeta > \mu$. Therefore, we can assume $r_1 = \frac{\zeta_0}{\mu_0} > \frac{[\text{cross-linker}]^2}{[\text{ATP.KSA}]}$ while computing the phase boundary. Since KSA concentration in the experiments ranges between $1 - 200nM$, PRC1 concentration ranges between $60 - 380nM$, and ATP concentration ranges between $1\mu M - 1mM$, [cross-linker] ranges between $1 - 580nM$, and [ATP.KSA] ranges between $0.02 - 190nM$. Therefore, $\frac{[\text{cross-linker}]^2}{[\text{ATP.KSA}]}$ ranges between $10^{-3}nM - 17mM$.

3.2.2 r_2

The nematic elasticity for microtubules of length $1.5\mu m$ in 3D is around $1.6 \times 10^{-9} N$ [18], and the shear modulus for microtubule networks in 3D is around $60Pa$[19]. Since the thickness of the sheet is around $80\mu m$, the 2D nematic elasticity, $K \sim 1.6 \times 10^{-9} N \times (80\mu m)$, and the 2D bending modulus, $\kappa \sim 60Pa \times (80\mu m)^3$. Thus, $r_2 = \frac{K_0}{\kappa_0} = \frac{K/l^3}{\kappa/([\text{cross-linker}]^2)} \sim \frac{1.6\times10^{-9} N/(1.5\mu m)^3}{60Pa\times(80\mu m)^2/([\text{cross-linker}]^2)}$, ranges between $10^{-3} - 415 nM^2\mu m^{-3}$. Therefore, we give r_2 a range of $10^{-4} - 10^3 nM^2\mu m^{-3}$ for the fitting.

3.2.3 r_3

$r_3 = \sqrt{\frac{K_0}{\zeta_0}} = \sqrt{\frac{K}{\zeta}}\sqrt{\frac{[\text{ATP.KSA}]}{l^3}}$; $\sqrt{\frac{K}{\zeta}}$ has units of length, and should range between the minimum (microtubule length $1.5\mu m$) and maximum (max channel length $\sim 3cm$) length scales in the system. Therefore, r_3 ranges between $1.5\mu m \times \sqrt{\frac{\min([\text{ATP.KSA}])}{\max(l)^3}} \sim 0.022 nM^{1/2}\mu m^{-1/2}$ to $3 \times 10^4 \mu m \times \sqrt{\frac{\max([\text{ATP.KSA}])}{\min(l)^3}} \sim 5.8 \times 10^5 nM^{1/2}\mu m^{-1/2}$

3.2.4 p_0

The minimum value of p_0 is zero, which corresponds to all PRC1 crosslinkers being inactive, and the maximum value of p_0 is 1, which corresponds to all PRC1 crosslinkers being actively crosslinking.

3.3 2D vs. 3D parameter values

Our 2D hydrodynamic model can be derived from a 3D model by integrating over the thickness, τ of the sheet. Then, the parameters in our 2D model are related to those of the corresponding 3D model by: $\mu = \tau\mu^{3D}$, $\zeta = \tau\zeta^{3D}$, $K = \tau K^{3D}$, and $\kappa = \tau\kappa^{3D}$. The parameter ratios ζ/μ, K/κ, K/ζ remain the same in 2D and 3D. Since we know η_{3D} from existing literature [19], we can compute ζ^{3D}, K^{3D}, and κ^{3D} from our fits for r_1, r_2, and r_3.

3.4 Estimation of μ_0

Given the parameters r_1, r_2, and r_3, prior knowledge of μ_0 allows us to infer the values of ζ_0, κ_0 and K_0. Recent bulk rheology experiments estimated the storage modulus G'=60Pa for a microtubule-kinesin network that ran out of ATP. In that case, the concentration of crosslinkers is equal to the initial concentration of molecular motors 120nM. We therefore estimate that $\mu_0 = 60Pa/(120nM)^2 = 4.17 \times 10^{15} Pa.M^{-2}$

3.5 Comparison of the value of ζ with values from the literature

Activity has been inferred previously by Ellis and co-workers in [18]. Briefly, they inferred the activity from tracking topological defects in 2D active nematics composed of microtubules and molecular motors. The value they measured is around 250mPa, which is about 3 orders of magnitude lower than the values reported here for the same concentrations of motors and ATP. There are a few differences between the two experiments that could explain this difference. First, Ellis et al. studied a 2D active nematic on an oil-water interface. Here, we have a suspension of microtubule bundles in 3D. Second, the local microtubule density is much larger in their study as they deplete all the microtubules on an oil-water interface. Lastly, we note that the activity has to be larger than the elasticity for any liquid like instability, which for the protein concentrations reported by Ellis [18] and by Gagnon in [19] mean $\zeta > \mu = 60Pa$

4 Materials and Methods

4.1 Protein purification protocols

4.1.1 Microtubules (MTs)

Tubulin dimers were purified from bovine brains through two cycles of polymerization - depolymerization in high molarity PIPES (1,4- piperazindiethanesulfonic acid) buffer [20]. Fluorophore-labeled tubulin was prepared by labeling the purified tubulin with Alexa-Fluor 647-NHS (Invitrogen, A-20006)[21]. GMPCPP (Guanosine 5'- (α, β methylenetriphosphate)), a non-hydrolyzable analogue of GTP was used to stabilize the dynamic instability in the MTs. Polymerization mixture consisted of 80μM tubulin (with 3% fluorescently labeled tubulin), 0.6mM GMPCPP and 1mM DTT (dithiothreitol) in M2B buffer (80mM PIPES, 1mM EGTA, 2mM $MgCl_2$). After adding all the components, the mixture was incubated at 37°C for 30 minutes, and subsequently for 6 hours at room temperature ($\sim$ 20°C). This method resulted in Microtubules of $\sim$ 1.5μm length [22]. The stock concentration was 8mg/mL. Microtubules were aliquoted in small volumes (10μL), flash-frozen in liquid nitrogen, and stored at -80°C.

4.1.2 Crosslinkers - PRC1-NSΔC

The truncated PRC1-NSΔC (MW : 58 kDa), a microtubule crosslinking protein, was expressed and purified in Rosetta BL21(DE3) cells using an established protocol described elsewhere [23]. The proteins were flash frozen with 40% sucrose and stored at -80°C. The final concentration of PRC1 was measured by a Bradford assay.

4.1.3 Molecular motors - K401 and K365 motors

K401-BIO-6xHIS (processive motor, dimeric MW-110 kDa) and K365-BIO-6xHIS (non-processive motor, MW-50 kDa) are biotinylated kinesin constructs derived from N-terminal domain of *Drosophila melanogaster* kinesin-1, truncated at residue 401 and 365, respectively, and labeled with six histidine tags. The motor proteins were transformed and expressed in Rosetta (DE3) pLysS cells and purified following established protocols described previously [24]. The proteins were stored in a 40% wt/vol sucrose solution at -80°C. The final concentration of kinesin was measured by Bradford assay.

We used tetrameric streptavidin (ThermoFisher, 21122, MW: 52.8 kDa) to assemble clusters of biotinlabeled kinesins (KSA). To make K401-streptavidin clusters, 5.7μL of 6.6μM streptavidin was mixed with 5μL of 6.4μM K401 and 0.5μL of 5mM DTT in M2B. This mixture was incubated on ice for 30 minutes. K365-streptavidin clusters were prepared by mixing, 5.7μL of 6.6μM streptavidin, 3.1μL of 20μM K365, 0.5μL of 5mM DTT and 1.94μL of M2B, and then left to incubate on ice for 30 minutes

4.1.4 Light activable motors: K365-iLID and K365-micro

K365-iLID and K365-micro were designed by Linnea Lemma and Tyler Ross and were purified following the protocol described in [25]. Briefly, two chimeras of the *Drosophila melanogaster* kinesin K-365, namely, K-365-iLID and K-365-micro, were expressed in Escherichia coli Rosetta 2(DE3)pLysS cells and purified using ÄKTA pure FPLC system. MBP domain was cleaved with TEV protease (Sigma Aldrich). The proteins were snap-frozen in 40% glycerol and stored at -80°C.

4.2 Assembling 3D active network

The networks are composed of:

- Alexa 647-labeled GMPCPP stabilized microtubules (MTs) with an exponential distribution of lengths with an average of 1.5μm, unless otherwise specified,

- Multi-motor kinesin complexes self-assembled from tetrameric streptavidin and two-headed biotinylated kinesin (K401-Bio) or single-headed biotinylated kinesin (K365-Bio) [26],
- a specific microtubule bundling protein, PRC1 (the protein regulator of cytokinesis 1), that passively crosslink antiparallel microtubules, but still allow interfilament sliding [23].

In the presence of ATP, these proteins form a network of extensile bundles. An ATP regeneration system (phosphoenol pyruvate (26mM PEP, Beantown Chemical, 129745) and pyruvate kinase/lactate dehydrogenase enzymes (2.8% v/v PK/LDH, Sigma, P-0294) was used to sustain a constant ATP concentration. An oxygen scavenging system comprised of glucose (18.7mM), DTT (5.5mM), glucose oxidase (1.4μM), and catalase (0.17μM) was used to decrease photobleaching. Active network composed of MTs (1.3mg/ml), ATP (1420μM) and K401 clusters (120nM) remain active for 6-8 hours. For some experiments (Fig. S3), PRC1 was replaced by either 20 kDa PEG (polyethylene glycol) (0.8% (wt/vol) [Sigma] or Pluronic F-127 2% (wt/vol) [F-127, Sigma P2443. MW: 12.5 kDa]. For consistency, a large volume of premix was prepared, aliquoted, and snap frozen in liquid nitrogen for each phase diagrams shown in Fig. 3. Frozen microtubules (stored at -80°C) were thawed immediately before use in an experiment. All the experiments were performed at room temperature.

4.3 Estimation of the ratio of motor clusters and crosslinkers per microtubules

For a microtubule of 1.5μm length, there are about 2444 tubulin dimers in its 13 protofilaments. If one tubulin dimer weighs 100kDa then one microtubule weighs $244,400$ kDa. Each active network contains 1.33mg/mL of microtubules which gives MT concentration of 5.45nM. Example: assuming all the clusters are bound to MTs, for [Motor cluster]= 120nM and [PRC1]=60nM, they are about 22 motor clusters and 11 crosslinkers per microtubules.

4.4 Controlling the length distribution of microtubules via end-to-end annealing

We control the length distribution of GMPCPP-stabilized, fluorescently labeled microtubules by end-to-end annealing at 37°C for different time duration (Fig. S10). The polymerization protocol described above produces MTs with an average length of 1.5μm at a tubulin concentration of 8mg/mL. Below, we describe how to increase the average microtubule length at a constant tubulin concentration. The slowly hydrolysable GTP analog, GMPCPP effectively reduces the microtubule dissociation [27], therefore, the increase in length can only be attributed to end-to-end joining and thus other mechanisms including dynamic instability, subunit exchange at the MT ends or nucleotide hydrolysis can be ignored [28]. Keeping the MT in the water bath at 37°C increase the average MT length (Fig. S3-d).

4.5 Flow chamber assembly

All flow chambers have the same dimensions (H=80μm, W=3mm) and were assembled using two different approaches. No differences were observed between the 2 methods. The first method relies on a microfabricated channel made out of PDMS. The channel was prepared from a molding master created by machining cyclic olefin copolymer (COC) [29]. PDMS was poured over the master, and cured for 1 h at 70°C. The channel was removed from the master, inlet and outlet were punched, followed by oxygen plasma bonding to a glass slide (26 $\times$ 75 $\times$ 1mm). The channel was incubated with 2% Pluronic solution for 30 minutes to block any non-specific protein adsorption. The second method consists in two glass slides spaced by a layer of parafilm. The glass surfaces were coated with an acrylamide brush to resist non-specific protein adsorption onto the glass due to depletion [30]. Parafilm spacers were cut and placed between the two glass surfaces followed by a mild heat treatment at 60°C to melt parafilm so it can bind to the glass surfaces. The active mixture was

loaded into each channel type by capillarity and sealed with an UV-curing optical adhesive (NOA 81, Norland Products Inc.).

4.6 Microscopy

4.6.1 Widefield microscopy

The Alexa 647 labeled microtubule networks were imaged using an inverted wide-field microscope (Nikon Ti-E or Ti2) with a fluorescent filter (Semrock Cy5-4040C), a SOLA light engine (Lumencor), a 10x objective (Nikon Pan Fluor, NA 0.3) and a CCD or a sCMOS camera (Andor Clara E, Hamamatsu orca flash 4.0). The illumination and the data acquisition were controlled by micro-manager (μManager, Version 2.0.0-gamma [31]). All the measurements were performed at room temperature.

4.6.2 Confocal microscopy

Confocal fluorescence images of microtubules were obtained under Leica Application Suite X (LAS X) control on a laser scanning confocal microscope (TCS-SP8, Leica Microsystems GmbH) equipped with photomultiplier tubes. Fluorescence was excited with $638nm$ laser diode for Alexa Fluor 647. For morphological analysis, a z-stack with a $775 \times 775\mu$m field of view was scanned with a step size of 2μm using a non-immersion 20x objective (HCX PL Fluotar, numerical aperture, NA = 0.50).

4.7 Light-activable motors: microscopy Protocol and light intensity measurement

We replace Kinesin-streptavidin clusters by light activable hetero-dimers K365-iLID and K365-micro. Active mixtures of microtubules were prepared with equimolar concentration (0.2μM) of K365-iLID and K365-micro, 30μM ATP, and the standard ATP regenerating system. The microtubule networks were imaged and photo-activated with an inverted Nikon Ti2 microscope equipped with 10X objective (Nikon Plan Apo λ 10X, NA 0.45), a SOLA light engine (Lumencor), and a sCMOS camera (Andor Zyla). The illumination and the data acquisition were controlled by micro-manager. All the measurements were performed at room temperature. Light activable motors were excited at $488nm$ and fluorescent images of Alexa647-labeled microtubules were acquired using a Cy5 filter. Only a predefined region of interest (ROI) of total field of view of the camera was illuminated. Typically, one experiment was run per sample. In all experiments, the input power, P_{input} of the blue light was measured with an optical power meter (PM100A, Thorlabs GmbH, Germany) at the sample plane at $488nm$. The total output power (P_{total}) on the sample surface from a blue light beam was calculated by:

$$P_{\text{total}} = P_{\text{input}} \cdot \frac{\text{Exposure time (s)}}{\text{time interval (s)}} \tag{21}$$

The illumination intensity (I) was calculated by:

$$I = \frac{P_{\text{total}}}{A} \tag{22}$$

where A is the area of the sample. Samples were prepared and flown into the microfluidic chambers in the dark.

4.8 Image Processing

4.8.1 Blur segmentation algorithm

The microtubule bundles are blurry when they buckle outside of the focal plane of the microscope. We developed an image analysis protocol to detect and segment such out-of-focus patches (Fig.

S2). We measured the magnitude of the gradient of the fluorescent intensity – aka the image sharpness. When the microtubules are out of focus, the sharpness is low. When they are in focus, the sharpness is large. The out-of-focus patches are usually large (characteristic size: 50-100μm), we therefore smoothed the sharpness to remove high frequency noise. Finally, we segmented the sharpness to identified the out-of-focus regions. All the pixels with a sharpness below a threshold value are considered out of focus, while all the others are in-focus. Overlay between the segmented out-of-focus patches and the original fluorescent images confirmed the effectiveness of this detection method. All the images were acquired with the same objective, exposure time, filter cube, light intensity and binning.

$$B = \frac{\text{Area of the out-of-focus patches}}{\text{Total area of the Field Of View}} \tag{23}$$

In Figure S4, we show that the bluriness is a robust method to detect the out of plane buckling. We compared various imaging settings: confocal vs widefield microscopy (S4-a), objectives with different Numerical Aperture (S4-b), Field of view of various sizes (S4-d) or objectives with different magnification and different N.A. (S4-e). The variations between all these imaging method was always smaller that the variation of the bluriness at different location in a single channel (Fig. S4-c)

4.8.2 In- plane flow measurement

In-plane flows (Fig. S1) were measured during the instability growth using MATLAB-based open-source particle image velocimetry (PIV) [32]. An interrogation window size of 64 pixels = 82.56μm was selected with 50% overlap.

4.8.3 Measurement of the in-plane instability wavelength

The wavelength of the in-plane instability was measured from heatmaps of the y-component of the velocity field using a custom-made Fast Fourier Transform (FFT) algorithm written in MATLAB (Fig. S8). FFT of the velocity profiles was computed along the initial axis of alignment. The resulting power spectrum revealed a strong periodic signal with a well-defined peak corresponding to a characteristic wavelength of in-plane instability.

4.8.4 Measurement of the out of plane instability wavelength

The wavelength of the instability was measured from a Fast Fourier Transform of the sharpness (Fig. S9, see section above about how to measure the sharpness). The wavelength of the out-of-plane buckling instability is equal to twice the periodicity of the sharpness as it does not differentiate between microtubule bundles above or below the focal plane.

4.8.5 Measurement of the microtubules' length distribution

To characterize the length distribution of the microtubules, we sampled the MT stock at a constant tubulin concentration (8mg/mL) after every 30 minutes over 5 hours of annealing at 37°C. The samples were diluted to 3000X with antioxidants, Trolox, and a 2.5% (w/v) solution of dextran (MW 500 kDa). The dilution helps to prevent overlaps of MTs that complicate automated filament recognition. For imaging fluorescently labelled microtubules, 5μL of the solution was placed between a coverslip and a coverslide. Imaging was performed on a standard fluorescence microscope (Nikon Eclipse Ti microscope) using a high numerical aperture oil objective (Nikon Plan Flour 100X/1.30). We wrote a custom Matlab script that quantifies the length distribution of MTs. Binary images of each MT were fitted with an ellipse. The long axis of the ellipse corresponds to the MT's length (Fig. S10).

5 Supplementary Figures

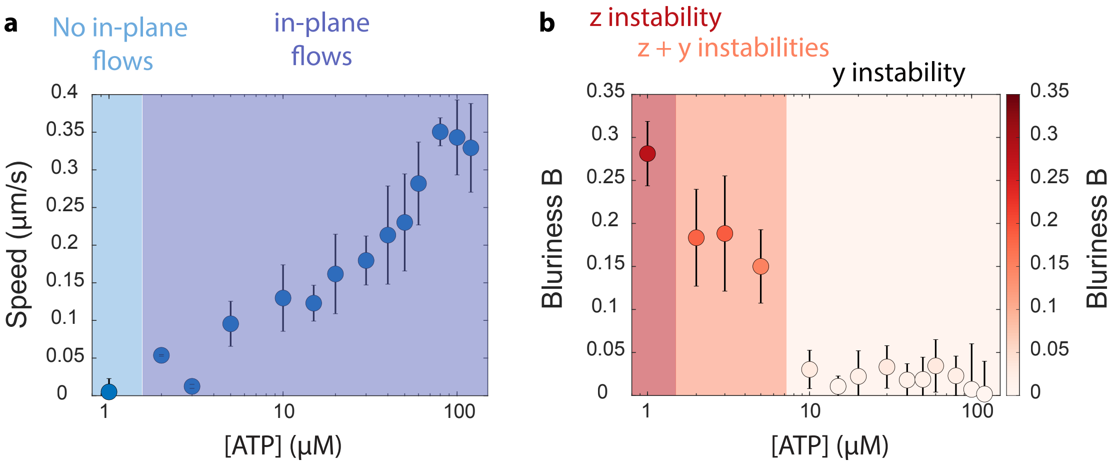

Fig. S1: **In-plane flows are suppressed at low ATP concentration**. a) in-plane flows measured by Particle Image Velocimetry and b) blurriness as a function of ATP concentration. No in-plane flows were observed for [ATP] $< 2\mu$M. Above that, the instability is a superposition of in-plane and out of plane deformations (2-5μM). Above 10μM, no deformations along the Z axis were detected. Error bars represent standard deviation of the mean over N=2 independent replicates. [motor clusters]$= 5nM$, [PRC1]$= 100nM$.

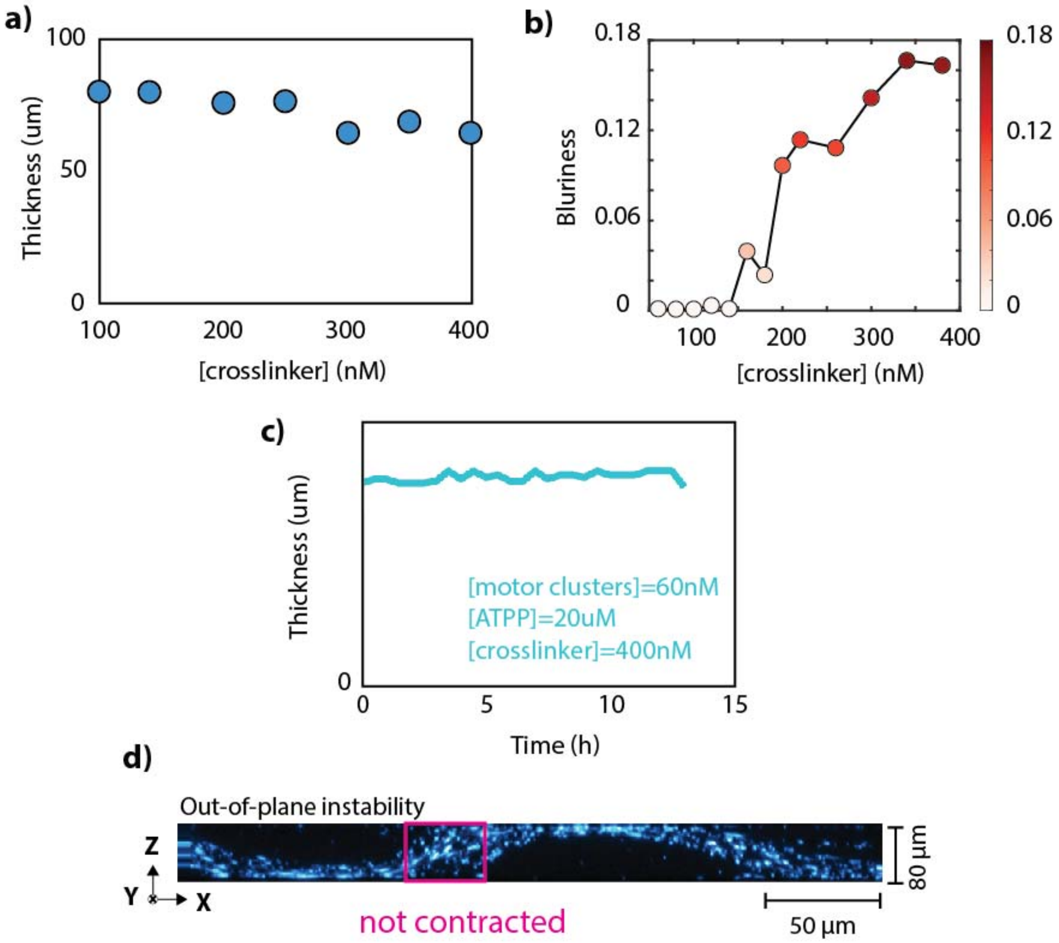

Fig. S2: **No anisotropic contraction along the Z-axis was detected for the range of molecular composition explored.** a) Thickness of the active sheet just before the instability. The range of crosslinkers explored here correspond to both in-plane instabilities c<200nM and out-of-plane buckling for c>200nM) as demonstrated in b), which shows the bluriness for the same range of molecular composition. c)Temporal evolution of the film thickness for an out-of-plane instability. No contraction is observed. d) One the nematic sheet buckles, the thickness changes as the film get pushed against the boundaries of the microfluidic channel. However, between two extremas, the material fills the entire channel, which proves that there is no anisotropic contraction along the Z axis. Experiments shown in d were respectively repeated N=5 times.

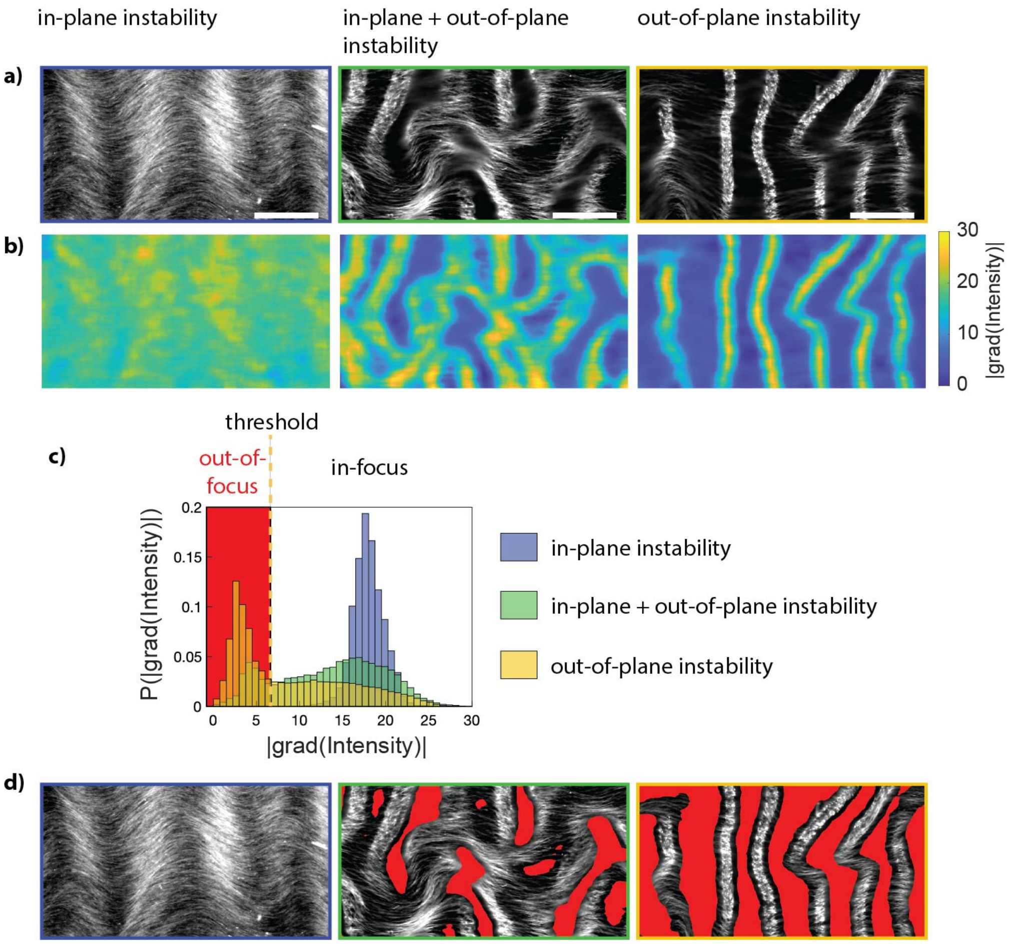

Fig. S3: **Method to detect if the network is bending in-plane or buckling out-of-plane.** a) Fluorescence images of the microtubule network (widefield fluorescent microscope, scale bar: 200μm). Three characteristic pictures are displayed showing an in-plane instability, an out-of-plane instability, or a superposition of both. b) heatmaps of the magnitude of the gradient of the fluorescent intensity. High values are associated with in-focus features, while low values are associated with out-of-focus features. c) probability distribution of the magnitude of the intensity gradient for the three heatmaps in b). Pixels with magnitude below a fixed threshold are considered out-of-focus. d) Original fluorescent images onto which the segmented out-of-focus patches have been highlighted in red. The pictures shown in d are the same as the ones shown in a and therefore have the same scale bar. Experiments shown in a, b, and d were for all the data collected in this manuscript.

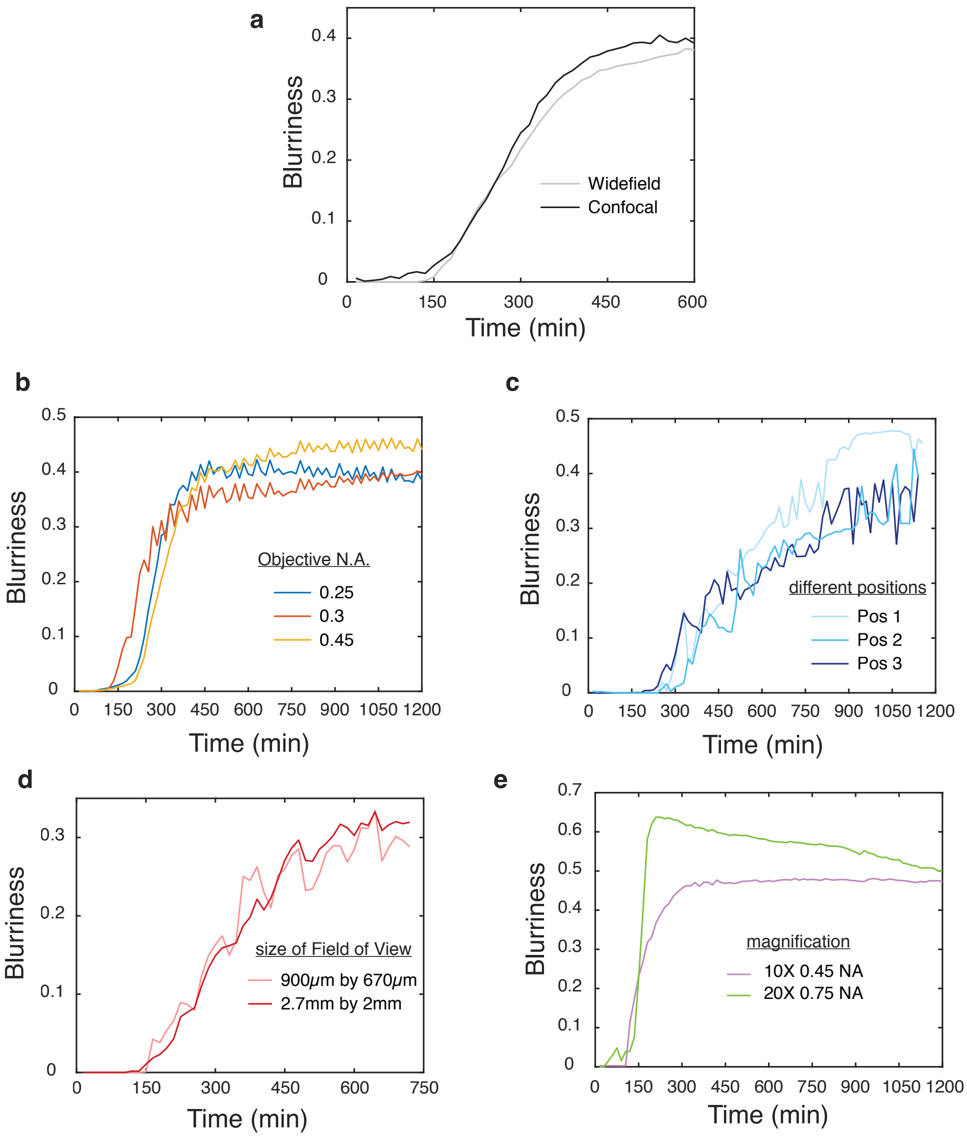

Fig. S4: **Blurriness detection and segmentation is robust to different imaging settings.** a) Confocal versus widefield fluorescent imaging, b) three 10X objectives with different numerical aperture, c) different positions within the same sample, d) different sized field of view taken with the same objective (1 vs 3 by 3 stitched), e) different magnification for same sized field of view

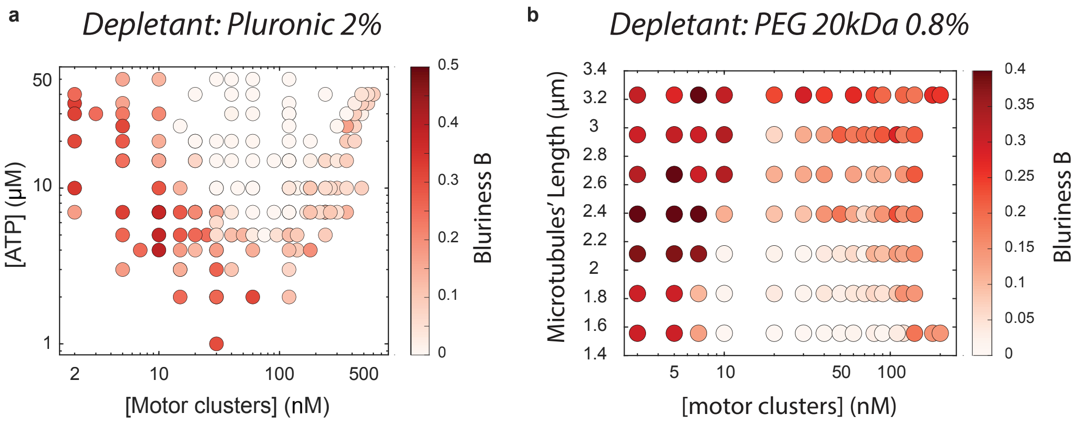

Fig. S5: **Increasing motor clusters concentration leads to a reentrant transition even when the crosslinker PRC1 is replaced by a non-specific bundler (2% Pluronic or 0.8% 20kDa PEG)**. a) ATP vs motor cluster concentrations phase diagrams show the re-entrant transition. b) Microtubules' length vs motor cluster concentrations. Color bar shows blurriness

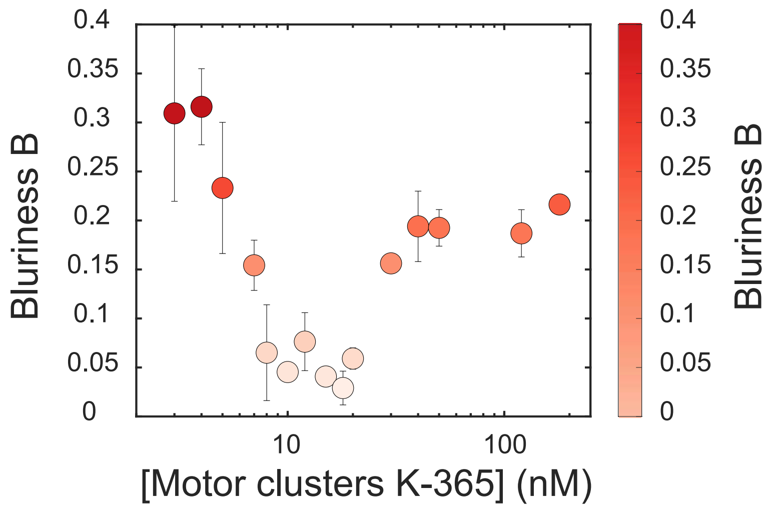

Fig. S6: **Non-processive K365 Kinesin motor clusters display the same re-entrant transition as the processive K401 motor clusters**. Color bar shows blurriness. Error bars represent standard deviation of the mean over N=3 independent replicates. [ATP]= 8μM, [PRC1]= $100nM$

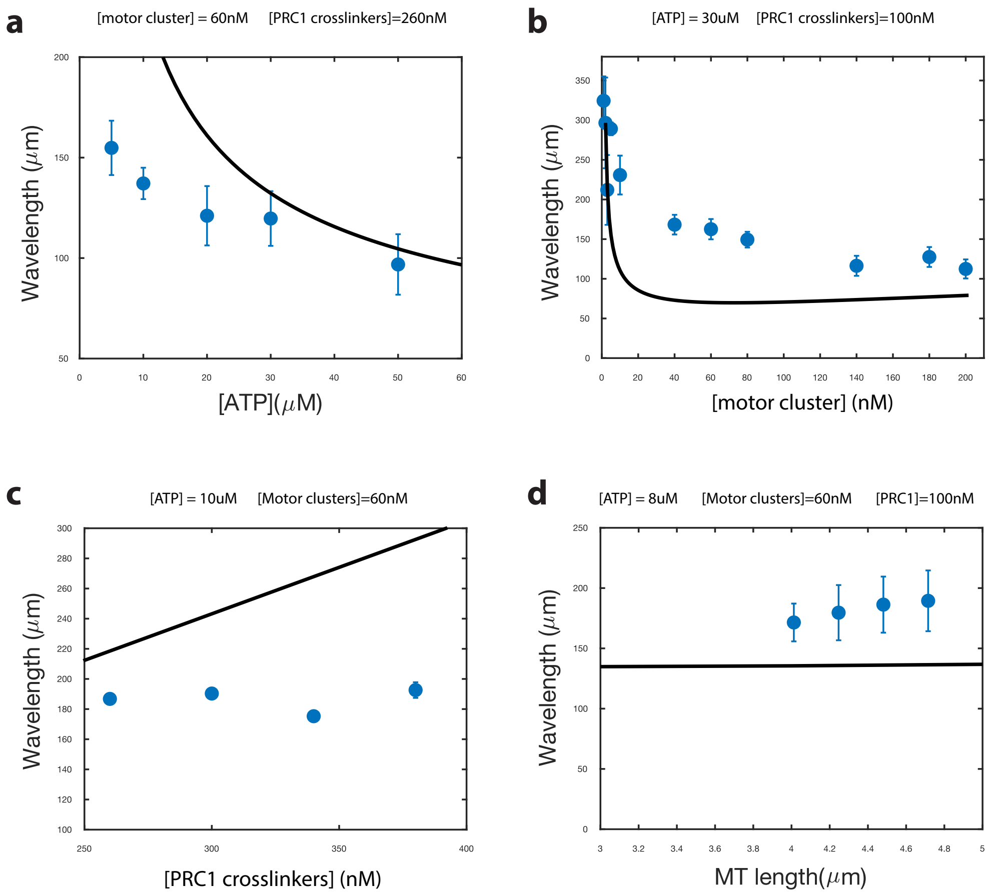

Fig. S7: **Comparison between theory and experiments for the out-of-plane wavelengths for various network compositions**: for varying a) ATP concentration, b) motor cluster concentration, c) PRC1 crosslinker concentration and d) MTs average length. Blue points: experiments, Black lines: theory. Error bars represent standard deviation of the mean over N=2 independent replicates.

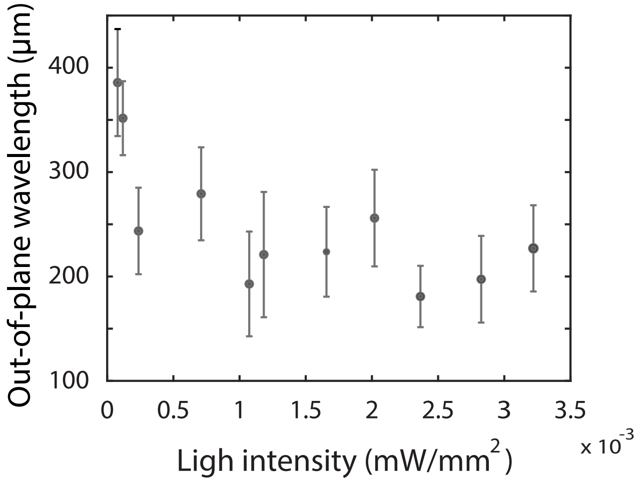

Fig. S8: **Out-of-plane wavelength for network composed of light-activable motors**. The wavelength decreases when the blue light intensity increases, which is compatible with an increase in activity. Error bars represent standard deviation of the mean over N=3 independent replicates. [ATP]=30uM, [LAMPS motors]=0.2uM.

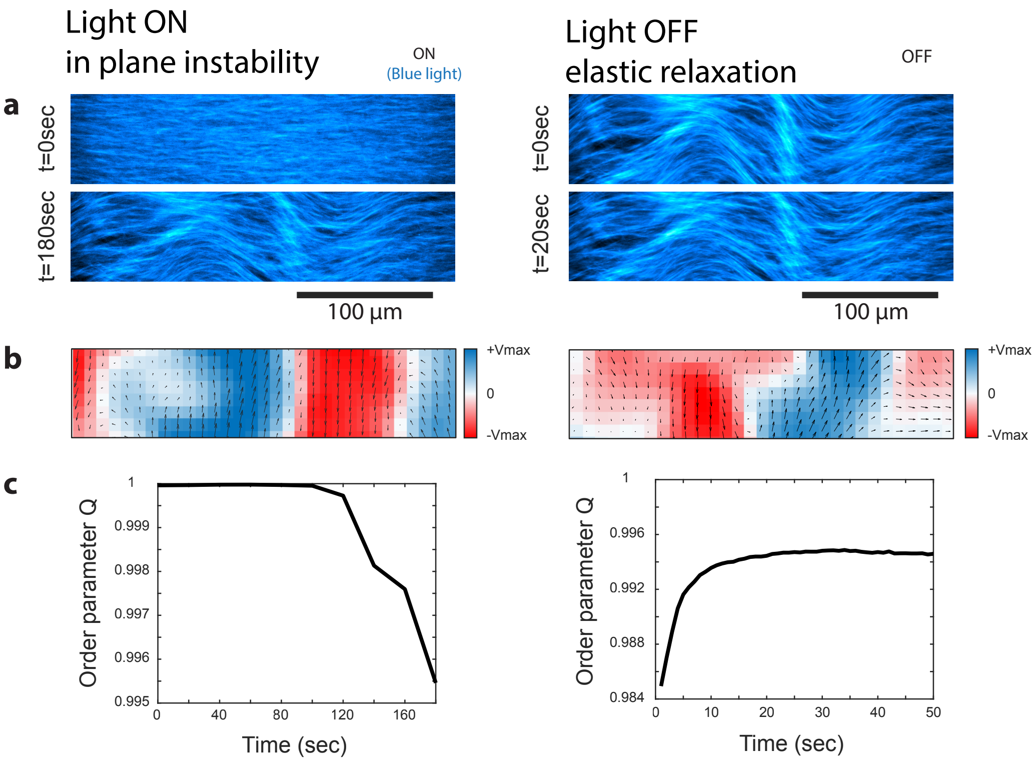

Fig. S9: **Turning off motor activity only allow a partial relaxation of the deformations**. Left column: active instability when blue light is one. Right column: elastic relaxation once the blue light is turned off. a) fluorescent image of the MT bundles. Experiments shown in a were respectively repeated N=3 times. b) velocity field overlaid onto the heatmap of the y-component of the velocity. C) time evolution of the order parameter that quantifies the nematic alignment of the MT bundles. When the light is on, the instability grows and Q decreases. When the light is off, the deformations partially relax, Q increases but do not reach Q=1.

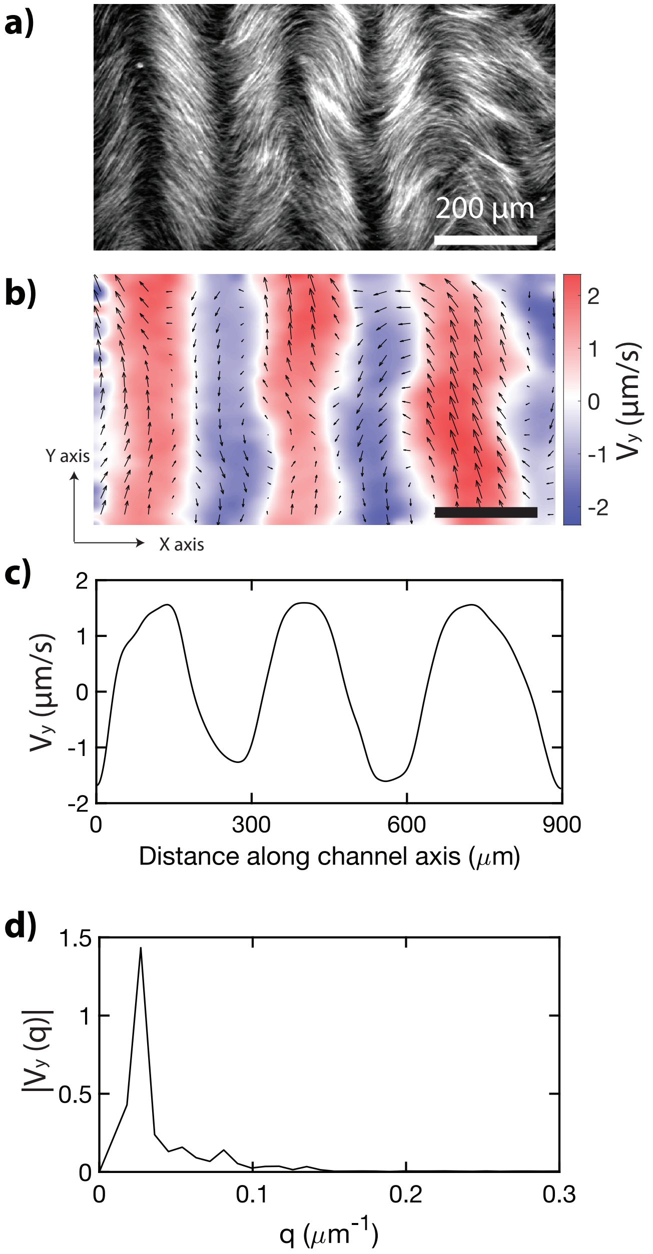

Fig. S10: **Characterization of the in-plane instability wavelength**. a) Fluorescent image of the microtubule bundles during the growth of the in-plane instability. b) velocity field (black arrows) overlaid onto the heatmap of the y-component of velocity field. c) Y-component of the velocity profile at fixed value of y, along the channel direction (x-axis). d) FFT spectrum of the corresponding velocity profile, q is the spatial frequency obtained by spatial domain of channel axis. λ is estimated by calculating the characteristic periodic length from the spectrum. Experiments shown in a were respectively repeated for all the data presented in figures S7 and S8.

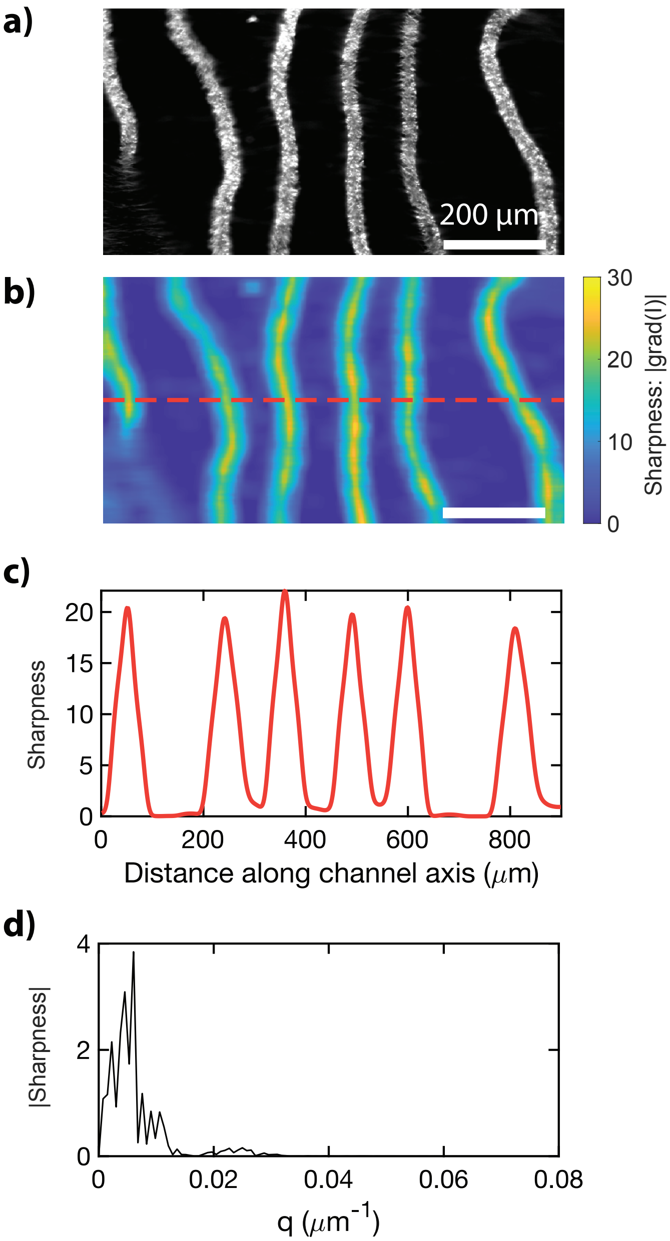

Fig. S11: **Characterization of the out-of-plane buckling wavelength**. a) fluorescent image of the microtubule bundles during the growth of the out-of-plane instability. b) Heatmap of the corresponding sharpness. c) A one-dimensional profile of the sharpness (at fixed value of y, red line shown in (b)) is used to calculate d) the power spectrum of the FFT. Experiments shown in a were respectively repeated for all the data presented in figures S7 and S8.

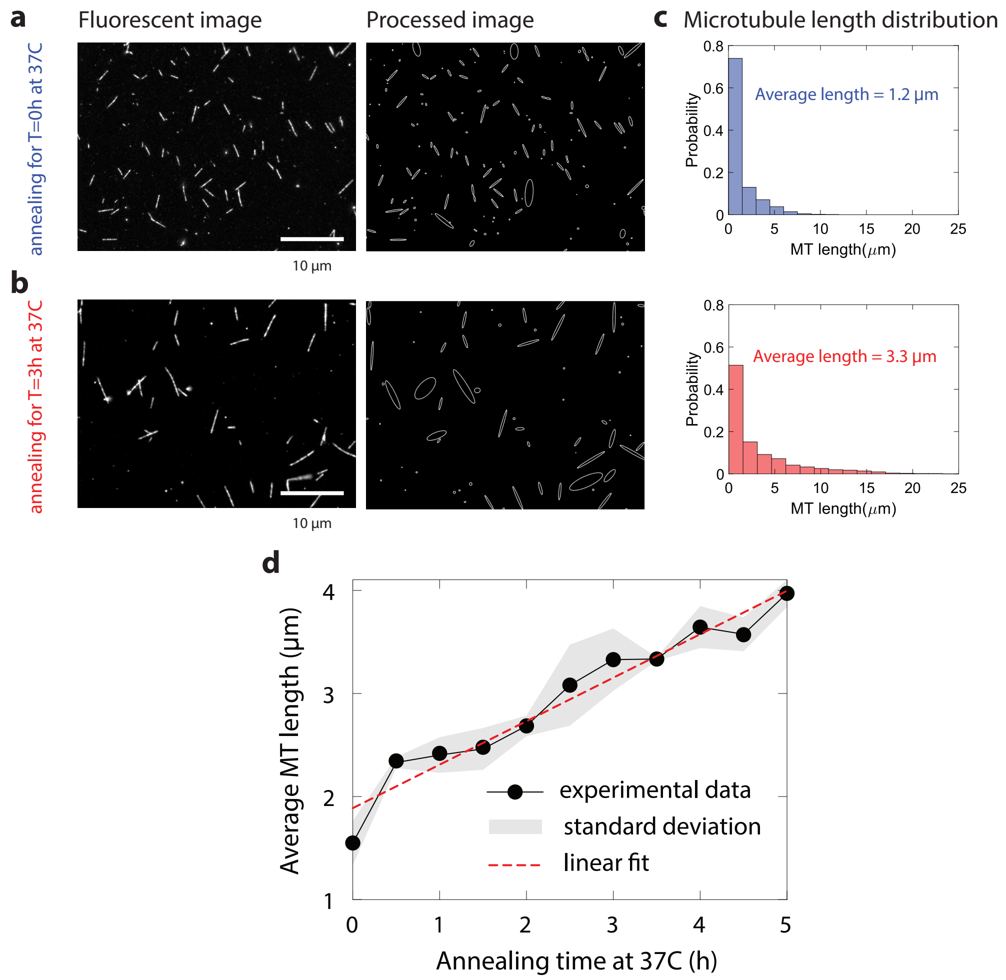

Fig. S12: **Controlling the length distribution of microtubules via end-to-end annealing**. a) Fluorescent images of the microtubules pre and post-annealing at 37°C. b) Ellipses are fitted onto the threshold fluorescent image to estimate the length distributions of the microtubules. c) Probability distributing of the microtubules' lengths before and after annealing for 3h at 37°C. The insert shows the averaged microtubule length. d) Mean MTs' length as a function of annealing time at 37°C. The black dotted line is the mean of the length distribution of the MTs and the grey area is the standard deviation in the experimental data over 3 realizations. The red dashed line is a linear fit. Experiments shown in a and b were respectively repeated a total of N=33 times.

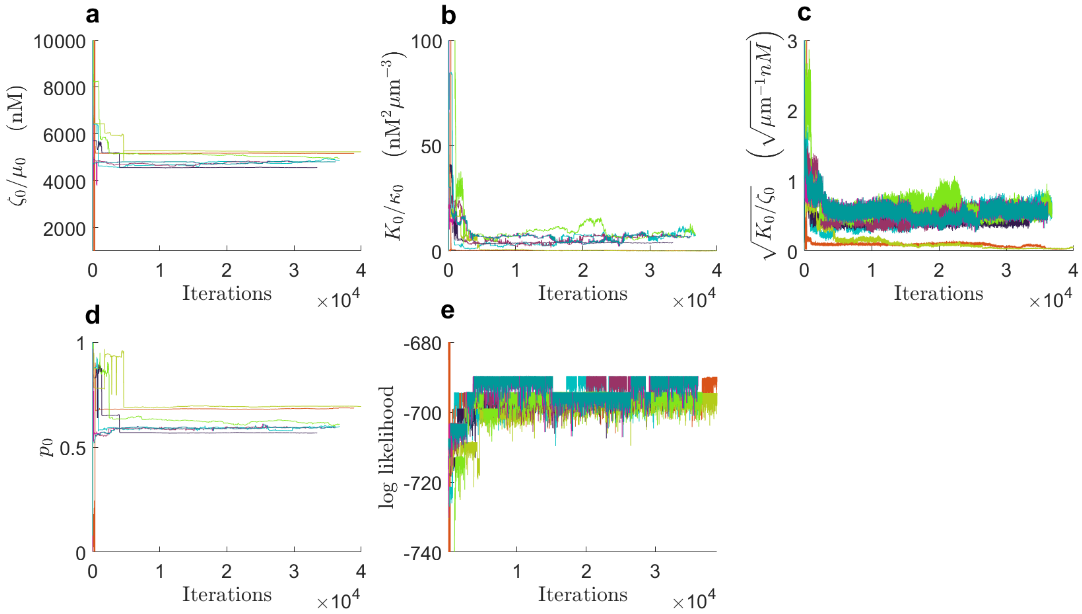

Fig. S13: **Parameter estimation through Markov Chain Monte Carlo**. Time series for the updated values of the fit parameters a) r1, b) r2, c) r3, and d) p0. We started 10 simulations from the upper end of the estimated parameter range, and another 10 from the lower end of the range. All the runs attained similar parameter values on convergence e) likelihood associated with the parameters displayed in a-d. Each colored curve corresponds to a different simulation. The parameter values we report are associated with the run that attained the largest log likelihood value on convergence (teal colored)

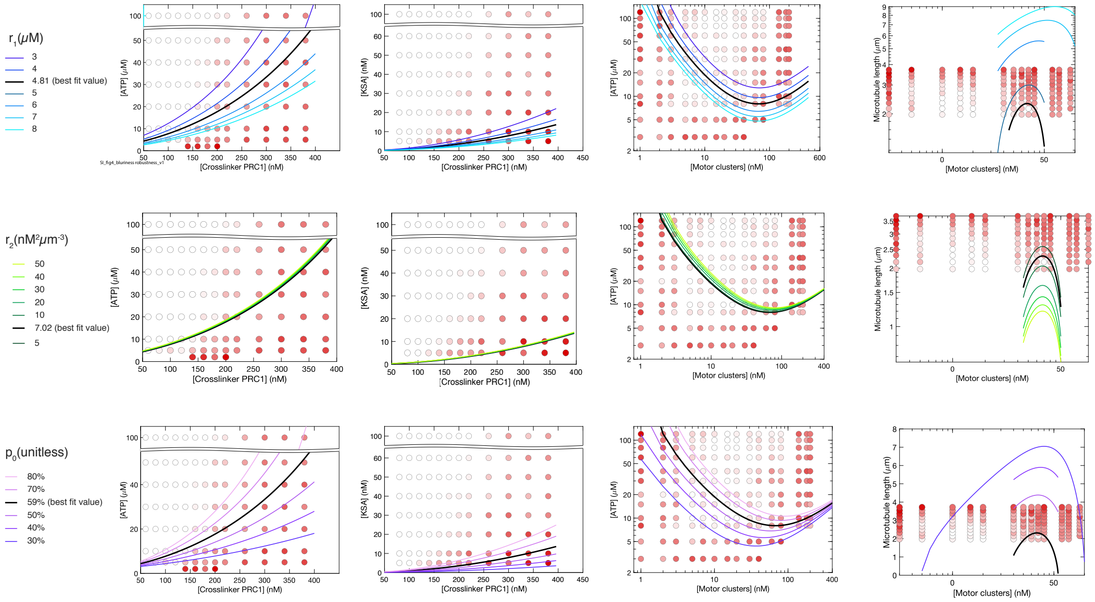

Fig. S14: **Robustness analysis of the theory/experiment comparison for various fit parameters**. We qualitatively compared the quality of the fit when the model parameters r1, r2 and p0 were varied around the best fit values. The experimental phase diagrams are the same as in figure 4 and the black line is the best theoretical fit. Most of the fits are robust to changes of model parameters, except the last phase diagram which depend on the length of the microtubules. This shows that this experimental parameter is not well taken into account in the hydrodynamic description.

6 Supplementary Video Captions

Video S1: In-plane bend instability of an aligned suspension of microtubules, crosslinkers and molecular motor clusters.

Widefield fluorescent microscope, 10X objective, [ATP]=50μM, [motor clusters]=20nM, [PRC1]=100nM.

Video S2: In-plane bend instability of an aligned suspension of microtubules, depletant, and molecular motor clusters.

Widefield fluorescent microscope, 4X magnification, [ATP]=1mM, [motor clusters]=20nM, [PEG 20kDa]=0.8% (vol/vol).

Video S3: Out-of-plane buckling of an aligned suspension of microtubules, crosslinkers and molecular motor clusters.

Widefield fluorescent microscope, [ATP]=10μM, [motor clusters]=10nM, [PRC1]=200nM.

Video S4: Out-of-plane buckling of an aligned suspension of microtubules, depletant, and molecular motor clusters.

Widefield fluorescent microscope, 10X magnification, [ATP]=10μM, [motor clusters]=20nM, [[PEG 20kDa]=0.8% (vol/vol).

Video S5: Confocal time-lapse imaging of the in-plane bend instability.

[ATP]=130μM, [motor cluster]=15nM, [PRC1]=50nM. Microtubule Length=1.5μm, Zstep 2.5μm, 1min23sec deltaT 20x

Video S6: Confocal time-lapse imaging of the out-of-plane buckling.

[ATP]=4μM, [motor cluster]=3nM, [PRC1]=100nM, Microtubule Length=2.3μm, Zstep size: 2μm, delatT 4min, 20X.

Video S7: Segmentation of the out-of-focus regions of interest.

First row: in-plane instability, second row: superposition of in-plan and out-of-plane deformations, third raw: out-of-plane buckling. The red areas correspond to the parts of the network that are out-of-focus.

Video S8: turning off motor activity only allow a partial relaxation of the deformations.

Fluorescent timelapse imaging of the microtubule bundles 30sec after blue light is turned off.

References

[1] P. G. d. Gennes and J. Prost, *The physics of liquid crystals.* Birman, J., series joint gen. ed. International series of monographs on physics, Oxford : New York: Clarendon Press ; Oxford University Press, 2nd ed. ed., 1998.

[2] T.-S. Nguyen and J. V. Selinger, "Theory of liquid crystal elastomers and polymer networks," *The European Physical Journal E*, vol. 40, p. 76, Sep 2017.

[3] R. Aditi Simha and S. Ramaswamy, "Hydrodynamic fluctuations and instabilities in ordered suspensions of self-propelled particles," *Phys. Rev. Lett.*, vol. 89, p. 058101, Jul 2002.

[4] T. Sanchez, D. T. N. Chen, S. J. DeCamp, M. Heymann, and Z. Dogic, "Spontaneous motion in hierarchically assembled active matter," *Nature*, vol. 491, no. 7424, pp. 431–434, 2012.

[5] S. P. Gilbert, M. R. Webb, M. Brune, and K. A. Johnson, "Pathway of processive atp hydrolysis by kinesin," *Nature*, vol. 373, pp. 671–676, Feb 1995.

[6] S. P. Gilbert, M. L. Moyer, and K. A. Johnson, "Alternating site mechanism of the kinesin atpase," *Biochemistry*, vol. 37, pp. 792–799, Jan 1998.

[7] S. P. Gilbert and K. A. Johnson, "Pre-steady-state kinetics of the microtubule-kinesin ATPase," *Biochemistry*, vol. 33, pp. 1951–1960, Feb. 1994.

[8] P. Xie, "Theoretical analysis of dynamics of kinesin molecular motors," *ACS Omega*, vol. 5, pp. 5721–5730, Mar 2020.

[9] M. L. Gardel, J. H. Shin, F. C. MacKintosh, L. Mahadevan, P. Matsudaira, and D. A. Weitz, "Elastic behavior of cross-linked and bundled actin networks," *Science*, vol. 304, no. 5675, pp. 1301–1305, 2004.

[10] Y. Luan, O. Lieleg, B. Wagner, and A. R. Bausch, "Micro- and macrorheological properties of isotropically cross-linked actin networks," *Biophysical Journal*, vol. 94, no. 2, pp. 688–693, 2008.

[11] O. Lieleg, M. M. A. E. Claessens, C. Heussinger, E. Frey, and A. R. Bausch, "Mechanics of bundled semiflexible polymer networks," *Phys. Rev. Lett.*, vol. 99, p. 088102, Aug 2007.

[12] B. Wagner, R. Tharmann, I. Haase, M. Fischer, and A. R. Bausch, "Cytoskeletal polymer networks: The molecular structure of cross-linkers determines macroscopic properties," *Proceedings of the National Academy of Sciences*, vol. 103, no. 38, pp. 13974–13978, 2006.

[13] Y.-C. Lin, G. H. Koenderink, F. C. MacKintosh, and D. A. Weitz, "Viscoelastic properties of microtubule networks," *Macromolecules*, vol. 40, no. 21, pp. 7714–7720, 2007.

[14] J. H. Shin, M. L. Gardel, L. Mahadevan, P. Matsudaira, and D. A. Weitz, "Relating microstructure to rheology of a bundled and cross-linked f-actin network in vitro," *Proceedings of the National Academy of Sciences*, vol. 101, no. 26, pp. 9636–9641, 2004.

[15] J. P. Straley, "Critical phenomena in resistor networks," *Journal of Physics C: Solid State Physics*, vol. 9, pp. 783–795, mar 1976.

[16] C. P. Broedersz, X. Mao, T. C. Lubensky, and F. C. MacKintosh, "Criticality and isostaticity in fibre networks," *Nature Physics*, vol. 7, pp. 983–988, 2011.

[17] E. E. Magat, "Liquid crystallinity in polymers, principles and fundamental properties, alberto ciferri, ed., vch, new york, 1991, 438 pp.," *Journal of Polymer Science Part A: Polymer Chemistry*, vol. 30, no. 5, pp. 955–955, 1992.

[18] P. W. Ellis, D. J. G. Pearce, Y.-W. Chang, G. Goldsztein, L. Giomi, and A. Fernandez-Nieves, "Curvature-induced defect unbinding and dynamics in active nematic toroids," *Nature Physics*, vol. 14, pp. 85–90, Jan 2018.

[19] D. A. Gagnon, C. Dessi, J. P. Berezney, R. Boros, D. T.-N. Chen, Z. Dogic, and D. L. Blair, "Shear-induced gelation of self-yielding active networks," *Phys. Rev. Lett.*, vol. 125, p. 178003, Oct 2020.

[20] M. Castoldi and A. V. Popov, "Purification of brain tubulin through two cycles of polymerization–depolymerization in a high-molarity buffer," *Protein Expression and Purification*, vol. 32, pp. 83–88, Nov 2003.

[21] G. Duclos, R. Adkins, D. Banerjee, M. S. Peterson, M. Varghese, I. Kolvin, A. Baskaran, R. A. Pelcovits, T. R. Powers, A. Baskaran, *et al.*, "Topological structure and dynamics of three-dimensional active nematics," *Science*, vol. 367, no. 6482, pp. 1120–1124, 2020.

[22] S. J. DeCamp, G. S. Redner, A. Baskaran, M. F. Hagan, and Z. Dogic, "Orientational order of motile defects in active nematics," *Nature materials*, vol. 14, no. 11, pp. 1110–1115, 2015.

[23] R. Subramanian, E. M. Wilson-Kubalek, C. P. Arthur, M. J. Bick, E. A. Campbell, S. A. Darst, R. A. Milligan, and T. M. Kapoor, "Insights into antiparallel microtubule crosslinking by prc1, a conserved nonmotor microtubule binding protein," *Cell*, vol. 142, no. 3, pp. 433–443, 2010.

[24] D. S. Martin, R. Fathi, T. J. Mitchison, and J. Gelles, "Fret measurements of kinesin neck orientation reveal a structural basis for processivity and asymmetry," *Proceedings of the National Academy of Sciences*, vol. 107, no. 12, pp. 5453–5458, 2010.

[25] T. D. Ross, H. J. Lee, Z. Qu, R. A. Banks, R. Phillips, and M. Thomson, "Controlling organization and forces in active matter through optically defined boundaries," *Nature*, vol. 572, no. 7768, pp. 224–229, 2019.

[26] P. Chandrakar, M. Varghese, S. Aghvami, A. Baskaran, Z. Dogic, and G. Duclos, "Confinement controls the bend instability of three-dimensional active liquid crystals," *Phys. Rev. Lett.*, vol. 125, p. 257801, Dec 2020.

[27] A. Desai, S. Verma, T. J. Mitchison, and C. E. Walczak, "Kin i kinesins are microtubule-destabilizing enzymes," *Cell*, vol. 96, no. 1, pp. 69–78, 1999.

[28] R. Williams and L. A. Rone, "End-to-end joining of taxol-stabilized gdp-containing microtubules," *Journal of Biological Chemistry*, vol. 264, no. 3, pp. 1663–1670, 1989.

[29] S. A. Aghvami, A. Opathalage, Z. Zhang, M. Ludwig, M. Heymann, M. Norton, N. Wilkins, and S. Fraden, "Rapid prototyping of cyclic olefin copolymer (coc) microfluidic devices," *Sensors and Actuators B: Chemical*, vol. 247, pp. 940–949, 2017.

[30] T. Sanchez, D. T. Chen, S. J. DeCamp, M. Heymann, and Z. Dogic, "Spontaneous motion in hierarchically assembled active matter," *Nature*, vol. 491, no. 7424, pp. 431–434, 2012.

[31] A. D. Edelstein, M. A. Tsuchida, N. Amodaj, H. Pinkard, R. D. Vale, and N. Stuurman, "Advanced methods of microscope control using Œºmanager software," *Journal of Biological Methods*, vol. 1, p. e10, Nov. 2014.

[32] W. Thielicke and E. J. Stamhuis, "Pivlab-time-resolved digital particle image velocimetry tool for matlab," *Published under the BSD license, programmed with MATLAB*, vol. 7, no. 0.246, p. R14, 2014.
