# Supplementary material for: Competing instabilities reveal how to rationally design and control active crosslinked gels
Source: Nat Commun. 2022 Oct 29;13:6465. doi: 10.1038/s41467-022-34089-9 (PMC9617906; doi:10.1038/s41467-022-34089-9)
Supplement: Supplementary file 3 — Description of Additional Supplementary Files [file 41467_2022_34089_MOESM3_ESM.pdf]

Description of Additional Supplementary Information Files Document

Supplementary Video Captions

Video S1: In-plane bend instability of an aligned suspension of microtubules, crosslinkers and molecular motor clusters.

Widefield fluorescent microscope, 10X objective, [ATP]= 50 µM, [motor clusters]= 20nM, [PRC1]= 100 nM.

Video S2: In-plane bend instability of an aligned suspension of microtubules, depletant, and molecular motor clusters.

Widefield fluorescent microscope, 4X magnification, [ATP]= 1 mM, [motor clusters]= 20 nM, [PEG 20kDa]=0.8 % (vol/vol).

Video S3: Out-of-plane buckling of an aligned suspension of microtubules, crosslinkers and molecular motor clusters.

Widefield fluorescent microscope, [ATP]= 10 µM, [motor clusters]= 10 nM, [PRC1]= 200 nM.

Video S4: Out-of-plane buckling of an aligned suspension of microtubules, depletant, and molecular motor clusters.

Widefield fluorescent microscope, 10X magnification, [ATP]= 10 µM, [motor clusters]= 20 nM, [PEG 20kDa]= 0.8 % (vol/vol).

Video S5: Confocal time-lapse imaging of the in-plane bend instability.

[ATP]= 130 µM, [motor cluster]= 15 nM, [PRC1]= 50 nM. Microtubule Length= 1.5 µm, Zstep size: 2.5 µm, 1 min 23 sec deltaT, 20x

Video S6: Confocal time-lapse imaging of the out-of-plane buckling.

[ATP]= 4 µM, [motor cluster]= 3 nM , [PRC1]= 100 nM, Microtubule Length= 2.3 µm, Zstep size: 2 µm, delatT 4 min, 20X.

Video S7: Segmentation of the out-of-focus regions of interest.

First row: in-plane instability, second row: superposition of in-plan and out-of-plane deformations, third raw: out-of-plane buckling. The red areas correspond to the parts of the network that are out-of-focus.

Video S8: turning off motor activity only allow a partial relaxation of the deformations.

Fluorescent timelapse imaging of the microtubule bundles 30 sec after blue light is turned off.
